# Hyperinsulinism-hyperammonemia syndrome due to an autosomal dominant *GLUD1* p.Ile497Met mutation

**DOI:** 10.1210/jcemcr/luag227

**Published:** 2026-08-20

**Authors:** Ayli S Anvaripour, Varuna Dhanabal, Ethan Jeffrey Blake, Alden F Dewey, Francesco Vetrini, Jamie Lynn Felton

**Affiliations:** Department of Pediatrics, Indiana University School of Medicine, Indianapolis, IN 46202, USA; Department of Pediatrics, Indiana University School of Medicine, Indianapolis, IN 46202, USA; Department of Pediatrics, Indiana University School of Medicine, Indianapolis, IN 46202, USA; Department of Pediatrics, Indiana University School of Medicine, Indianapolis, IN 46202, USA; Department of Pediatrics, Division of Pediatric Endocrinology, Riley Hospital for Children at Indiana University Health, Indianapolis, IN 46202, USA; Division of Clinical Genetics and Molecular Biology, Indiana University, Indianapolis, IN 46202, USA; Department of Pediatrics, Indiana University School of Medicine, Indianapolis, IN 46202, USA; Department of Pediatrics, Division of Pediatric Endocrinology, Riley Hospital for Children at Indiana University Health, Indianapolis, IN 46202, USA

**Keywords:** hyperinsulinism-hyperammonemia (HI/HA) syndrome, hypoglycemia, hyperammonemia

## Abstract

Hyperinsulinism-hyperammonemia (HI/HA) syndrome is a rare metabolic disorder caused by mutations in *GLUD1*, which encodes the mitochondrial enzyme glutamate dehydrogenase (GDH). This condition is characterized by recurrent hypoglycemia, elevated plasma ammonia, and potential neurological complications. We report the case of a 12-month-old male who presented with episodic hypoglycemia, hyperammonemia, and seizures. Family history was notable for childhood-onset hypoglycemia and seizures in the patient's mother and sister. The patient's laboratory findings during hospitalization were significant for markedly elevated C-peptide level of 4.9 ng/mL (SI: 1.63 nmol/L) (reference range; 1.1-4.4 ng/mL [SI: 0.36-1.46 nmol/L]). Insulin was inappropriately detectable at 20.2 µIU/mL (SI: 124.92 pmol/L) during hypoglycemia (reference range; 4.00-30.00 µIU/mL [SI: 24.0-180.0 pmol/L]). Genetic testing identified c.1491A>G (p.Ile497Met) in *GLUD1*. Following initiation of diazoxide therapy, he experienced symptomatic improvement with a marked reduction in hypoglycemic episodes. This case expands the literature on autosomal dominant HI/HA syndrome associated with an inherited *GLUD1* p.Ile497Met mutation.

## Introduction

Congenital hyperinsulinism (CHI) is the most common cause of persistent hypoglycemia in infancy [1]. It is associated with several genetic mutations, most notably *ABCC8* and *KCNJ11* defects affecting the pancreatic β-cell K_ATP_ channel [1, 2]. One subset of CHI is hyperinsulinism-hyperammonemia (HI/HA) syndrome, which most commonly results from gain-of-function mutations in the mitochondrial enzyme, glutamate dehydrogenase (GDH), encoded by the *GLUD1* gene that regulates insulin secretion from pancreatic β-cells [1]. Hyperinsulinism-hyperammonemia syndrome displays clinical features spanning from recurrent hypoglycemia with hyperammonemia to neurodevelopmental impairment and seizures [2, 3]. This condition differs from K_ATP_ channel hyperinsulinism in that HI/HA typically presents with postprandial, protein-sensitive hypoglycemia and demonstrates responsiveness to diazoxide [1, 4]. Most cases of HI/HA result from de novo mutations in the *GLUD1* gene [2, 3]. An individualized treatment approach is recommended for patients with hyperinsulinism [5]. We report the case of a 12-month-old male with recurrent hypoglycemia and a family history of hypoglycemia and seizures, who exhibited clear clinical improvement with diazoxide therapy. Although the *GLUD1* p.Ile497Met mutation has been previously reported as a de novo mutation in the literature, this case adds to the limited literature describing autosomal dominant HI/HA with a *GLUD1* p.Ile497Met mutation [6, 7].

## Case presentation

A 12-month-old male was hospitalized for evaluation of new-onset seizures. He was seen at an outside hospital emergency department after 2 episodes of generalized shaking followed by several minutes of unresponsiveness. His initial blood glucose was 60 mg/dL (SI: 3.3 mmol/L) (reference range; 70-99 mg/dL [SI: 3.9-5.5 mmol/L]). Complete blood count, basic metabolic panel, and urinalysis were unremarkable. The patient had no prior history of similar episodes and had been feeding normally, having consumed a bottle of whole milk and a snack shortly before the hypoglycemic episode.

His medical history was notable for global developmental delay. He was born at 31 weeks' gestation via cesarean delivery following a pregnancy complicated by diet-controlled gestational diabetes. The patient was large for gestational age, with a birth weight of 2025 g (95th percentile). His 1-month neonatal intensive care unit (NICU) course was uncomplicated, and there were no documented episodes of hypoglycemia during his NICU stay. All newborn screening results were normal. Diet consisted of transitional infant formula until 4.5 months, followed by introduction of pureed foods, with higher-protein foods added at 9 months and whole cow's milk at 10 months. No recent dietary changes were reported prior to admission. Family history was significant for episodic hypoglycemia and childhood-onset epilepsy in the patient's mother and sister, with seizures occurring beyond the neonatal period. It is unclear whether glucose levels were assessed at the time of their seizures. The mother reported that her symptoms resolved with age. The patient's neurologic exam was positive for central hypotonia with ankle spasticity. The remainder of his physical examination was normal. Weight on admission was 7.96 kg (*z* = −1.78).

## Diagnostic assessment

Within 12 hours of admission, despite adequate oral intake, the patient developed hypoglycemia with a glucose of 46 mg/dL (2.6 mmol/L). Given recurrent hypoglycemia, a diagnostic fast was initiated, during which the patient's blood glucose did not fall below 50 mg/dL (2.8 mmol/L) until 11 hours into the fast. Blood glucose transiently normalized following administration of a carbohydrate-containing snack but decreased to <50 mg/dL (2.8 mmol/L) within 4 hours. A critical sample, defined as diagnostic laboratory studies obtained during hypoglycemia and before treatment to identify the underlying etiology, was collected during a subsequent fed-state hypoglycemic episode approximately 12 hours after the prior episode (Table 1). Serum blood glucose was 28 mg/dL (1.56 mmol/L). C-peptide was 4.9 ng/mL (SI: 1.63 nmol/L) (reference range; 1.1-4.4 ng/mL [SI: 0.36-1.46 nmol/L]). Insulin was inappropriately detectable at 20.2 µIU/mL (SI: 124.92 pmol/L) during hypoglycemia (reference range; 4.00-30.00 µIU/mL [SI: 24.0-180.0 pmol/L]). Appropriate growth hormone and cortisol responses were observed following glucagon stimulation. Plasma ammonia, obtained outside of the hypoglycemic episode, was elevated at 1.31 mg/dL (SI: 116 µmol/L) (reference range; 0.067–0.53 mg/dL [SI: 6-47 µmol/L]). Ammonia levels in HI/HA syndrome are typically chronically elevated and need not be obtained during hypoglycemia. The remainder of the laboratory findings were unremarkable (Table 2).

**Table 1:** Critical sample obtained during admission

Test	Result	Reference range
C-peptide	4.9 ng/mL (1.63 nmol/L)	1.1-4.4 ng/mL (0.36-1.46 nmol/L)
Insulin	20.82 µIU/mL (124.92 pmol/L)	4.00-30.00 µIU/mL (24.0-180.0 pmol/L)
β-Hydroxybutyrate	0.15 mg/dL (1.55 mmol/L)	0.02-0.27 mg/dL (0.21-2.81 mmol/L)
Growth hormone	2.256 ng/mL (1.02*10^−7^ mmol/L)	NA
Cortisol	15.0 µg/dL (414 nmol/L)	AM: 8-25 µg/dL (220.72-690 nmol/L) PM: 4-20 µg/dL (110.4-552 nmol/L)
Free fatty acids	0.31 mmol/L	≤0.99 mmol/L
Serum glucose	28 mg/dL (1.56 mmol/L)	70-99 mg/dL (3.9-5.5 mmol/L)
Lactate	18.9 mg/dL (2.1 mmol/L)	4.5-19.8 mg/dL (0.5-2.2 mmol/L)
Ammonia	1.31 mg/dL (116 µmol/L)	0.067-0.53 mg/dL (6-47 µmol/L)

Values in parentheses are in Système International (SI) units.

Abbreviation: NA, not available.

**Table 2:** Serum chemistry and liver function

Test	Result	Reference range
Sodium	136 mEq/L (136 mmol/L)	135-145 mEq/L (135-145 mmol/L)
Potassium	4.1 mEq/L (4.1 mmol/L)	3.5-5.5 mEq/L (3.5-5.5 mmol/L)
Chloride	104 mEq/L (104 mmol/L)	98-108 mEq/L (98-108 mmol/L)
Bicarbonate	21 mEq/L (21 mmol/L)	22-29 mEq/L (22-29 mmol/L)
Anion gap	11 mEq/L (11 mmol/L)	3-11 mEq/L (3-11 mmol/L)
BUN	11 mg/dL (3.93 mmol/L)	5-20 mg/dL (1.79-7.14 mmol/L)
Creatinine	< 0.20 mg/dL (< 17.7 µmol/L)	0.20-0.70 mg/dL (17.7-61.9 µmol/L)
Calcium	9.9 mg/dL (2.475 mmol/L)	8.5-10.5 mg/dL (2.125-2.625 mmol/L)
AST	35 U/L	13-39 U/L
ALT	26 U/L	7-52 U/L
Alkaline phosphate	175 U/L	65-380 U/L
Total bilirubin	0.2 mg/dL (3.42 µmol/L)	0.0-1.0 mg/dL (0.0-17.1 µmol/L)
Direct bilirubin	< 0.1 mg/dL (< 1.71 µmol/L)	0.0-0.2 mg/dL (0.0-3.42 µmol/L)
Total protein	6.7 g/dL (67 g/L)	6.4-8.0 g/dL (64-80 g/L)
Albumin	4.0 g/dL (40 g/L)	3.1-4.7 g/dL (31-47 g/L)

Values in parentheses are in Système International (SI) units.

Abbreviations: ALT, alanine aminotransferase; AST, aspartate aminotransferase; BUN, blood urea nitrogen.

Neurologic evaluation included an electroencephalogram (EEG), which demonstrated occasional, poorly formed bifrontal spike-wave discharges. Fast-spin brain magnetic resonance imaging revealed mild white matter volume loss and ventriculomegaly, associated with prematurity.

Clinical rapid whole-genome sequencing identified a maternally inherited heterozygous *GLUD1* c.1491A>G (p.Ile497Met) mutation, which was not identified in available genomic databases and was classified as likely pathogenic based on prior reports of *GLUD1*-related HI/HA syndrome [6, 7]. In an early publication, the de novo *GLUD1* c.1491A>G (p.Ile497Met) mutation (previously referred to as Ile444Met) was identified in a 15-month-old female presenting with seizures. The patient's lymphoblasts exhibited reduced GDH activity and allosteric response relative to controls. Functional studies demonstrated that the inhibitory effects of guanosine triphosphate (GTP) were significantly reduced in cells transfected with the construct expressing the mutation compared with control complementary deoxyribonucleic acid, indicating impaired GTP-mediated regulation of GDH activity [6].

## Treatment

The patient was started on diazoxide 40 mg (10 mg/kg/day) orally twice daily and chlorothiazide 40 mg (10 mg/kg/day) orally every 12 hours. Blood glucose levels stabilized over a 48-hour period. He successfully completed a 12-hour fasting challenge without hypoglycemia. Given his abnormal EEG findings, the patient was started on levetiracetam 150 mg twice daily per neurology recommendations.

## Outcome and follow-up

The patient was discharged on diazoxide, chlorothiazide, and levetiracetam, with both neurology and endocrinology follow-up. He was continued on levetiracetam 150 mg twice daily by neurology. Diazoxide therapy has been maintained with ongoing endocrinology follow-up. The patient is currently 22 months old. The patient's mother declined genetic testing for the patient's sister.

## Discussion

Hyperinsulinism-hyperammonemia syndrome is a rare form of CHI caused by gain-of-function mutations in *GLUD1*, located on chromosome 10q23.3, which encodes glutamate dehydrogenase (GDH) [1]. Glutamate dehydrogenase activity is enhanced by leucine via allosteric activation and suppressed by inhibition from GTP. Hyperinsulinism-hyperammonemia syndrome was described in 1996 by Zammarchi et al [4] who reported a family with postprandial hypoglycemia and hyperammonemia. Stanley et al [3] later proposed *GLUD1* mutations of GDH as the underlying cause of HI/HA in 8 individuals.

Over the past 2 decades, the number of reported HI/HA mutations has increased substantially, with up to 80% of cases attributable to de novo mutations [2]. Mutations in exons 6 and 7 disrupt GTP binding directly, whereas mutations in exons 11 and 12 alter the antenna region, a unique allosteric domain, and impair GTP-mediated inhibition [8, 9]. Nevertheless, mutations in both regions are generally associated with reduced sensitivity to GTP-mediated inhibition [10]. Some studies have linked *GLUD1* mutations in exons 6 and 7 to an increased risk of epilepsy, although this association has not been consistently observed, and while 1 study reported a significant association between mutations in exons 11 and 12 and neurologic disorders, this finding has not been supported by other studies [11, 12]. Small sample sizes may have limited the ability to detect associations. Greater preservation of sensitivity to GTP inhibition has been associated with milder hypoglycemia [3]. In enzymatic studies, both de novo and familial GDH mutations demonstrated impaired GTP inhibition, with more pronounced impairment often observed in sporadic mutations, contributing to increased enzyme activity [3].

A small number of cases involving de novo *GLUD1* p.Ile497Met mutations have been previously reported in the literature. Aso et al [6] reported the p.Ile497Met mutation (previously known as p.Ile444Met) in exon 11 of *GLUD1* in a 13-year-old Japanese patient experiencing absence seizures with hypoglycemia and hyperammonemia. The patient experienced new-onset afebrile seizures at 15 months of age which were refractory to antiepileptic medications. The patient's mutation exhibited disrupted allosteric regulation of GDH, resulting in decreased sensitivity of the enzyme to GTP inhibition [6]. No intellectual disability was reported. Corrêa-Giannella et al [7] reported a c.1491A>G (p.Ile497Met) mutation in a 6-year-old Brazilian girl presenting with fasting hypoglycemia and hyperammonemia, generalized tonic-clonic seizures beginning at 7 months of age that were unresponsive to anticonvulsants, and significant cognitive impairment. Both cases were de novo mutations diagnosed later in childhood and adolescence. Both patients responded to diazoxide therapy and dietary modification. It is unclear whether hypoglycemia was present during the seizure episodes in these patients.

Beyond establishing inheritance, this case highlights notable phenotypic variation across affected family members, including differences in age of onset, neurological involvement, and apparent attenuation of symptoms with age in the proband's mother. The delayed presentation of HI/HA syndrome in late infancy, potentially precipitated by increased dietary protein intake, underscores the importance of considering this diagnosis in older infants without a neonatal history and of initiating timely treatment to prevent neuroglycopenic injury. In prior case reports of this mutation, hypoglycemia may have gone unrecognized due to lack of glucose testing, potentially contributing to delayed diagnosis and apparent poor response to anticonvulsant therapy. Although hypoglycemia in HI/HA typically presents during infancy, diagnosis may be delayed because affected individuals may exhibit relatively mild fasting intolerance compared with other forms of CHI.

The pathogenesis of epilepsy and neurodevelopmental delay in HI/HA syndrome remains incompletely understood. Seizures may occur in the absence of hypoglycemia, suggesting that neurologic manifestations may reflect mechanisms beyond hypoglycemia alone [13]. Supporting this hypothesis, a 2022 glutamate-weighted chemical exchange saturation transfer MRI (GluCEST MRI) study demonstrated greater variability in hippocampal glutamate signals among individuals with HI/HA compared with healthy controls, suggesting altered cerebral glutamate homeostasis as a potential contributor to the neurologic phenotype [14].

Diazoxide remains the first-line therapy for HI/HA, and chlorothiazide is often co-administered to mitigate fluid retention [1]. Some mutations have been managed with dietary modification alone such as limiting protein intake, others with diazoxide, and more severe cases have required subtotal pancreatectomy [3]. Compared with the previously reported de novo case of the Brazilian child with the same mutation, who developed seizures at 7 months and exhibited severe intellectual disability, our patient demonstrated a later seizure onset at 12 months of age and milder neurodevelopmental involvement. The comparatively milder presentation raises the possibility of a more favorable clinical trajectory, similar to his affected family members. This report highlights autosomal dominant inheritance, variable expressivity, and potential age- and environment-dependent disease modulation associated with the *GLUD1* p.Ile497Met mutation. Additional longitudinal studies are needed to clarify genotype-phenotype correlations and improve prognostication and management of HI/HA syndrome.

## Learning points

- Hyperinsulinism-hyperammonemia syndrome should be considered in toddlers presenting with recurrent hypoglycemia, particularly in the setting of seizures and developmental delay.
- Early recognition and treatment of HI/HA syndrome are critical to prevent neuroglycopenic complications and support normal neurodevelopment.
- Diazoxide remains the primary treatment for HI/HA syndrome, although spontaneous resolution of hypoglycemia may occur, and a trial off diazoxide may be considered.

## Contributors

All authors made individual contributions to authorship. A.S.A. was responsible for drafting all sections of the manuscript. V.D. contributed significantly to the literature review, drafting of the introduction and discussion. A.S.A., V.D., E.J.B., A.F.D., F.V., and J.L.F. were involved in the diagnosis and management of this patient and manuscript revision. All authors reviewed and approved the final draft.

## Data Availability

Data sharing does not apply to this article as no datasets were generated or analyzed during the current study.
